# The Role of Culture in the Justification and Perpetuation of Domestic Violence: The Perspectives of Service Providers in Kyrgyzstan

**DOI:** 10.1177/10778012231186814

**Published:** 2023-07-24

**Authors:** Saltanat Childress, Nibedita Shrestha, Kanykei Kenensarieva, Jildyz Urbaeva, Rachel Voth Schrag

**Affiliations:** 1 University of Texas-Arlington School of Social Work, Arlington, TX, USA; 2 47764 American University of Central Asia, Bishkek, Kyrgyzstan; 3 University at Albany School of Social Welfare, Albany, NY, USA

**Keywords:** Kyrgyzstan, Central Asia, domestic violence, cultural justification of violence, service providers’ perspectives

## Abstract

The study explores the perspectives of service providers on cultural and social reasons used to justify domestic violence in Kyrgyzstan. Results indicate that cultural norms, notably patriarchal customs, immense pressure put on women to save the marriage, stigma of divorce, low status assigned to women, wide acceptance of violence as natural, and fear of retaliation were major reasons that perpetuated domestic violence. Scholars, policymakers, and service providers must collaborate to actively dispel widely accepted beliefs about gender, marriage, and women's status, and to break the cycle of abuse providing help at the individual and community levels.

## Background

Domestic violence (DV; also known as Intimate Partner Violence or IPV^1^) is a major public health concern and a violation of human rights that disproportionately affects women (CDC, 2021; WHO, 2005). Global estimates indicate that one-third of all women who have been in a relationship have experienced physical and/or sexual violence (WHO, 2013). Violence has been shown to adversely affect women's health, with evidence of an increased risk of HIV/AIDS, mortality, and a range of reproductive, mental, and physical health outcomes (Cambpell, 2002; Heise et al., 1994). In addition to these debilitating effects on physical, mental, and reproductive health, DV has enormous social economic costs and negative multiplier effects throughout society (Day, 1995; Duvvury et al., 2013; UNDP, 2013). The World Health Organization (2005) conducted a review of the literature and concluded that interpersonal violence is expensive; that the economic costs are largely borne by the public sector; and that costs associated with interpersonal violence place a disproportionately heavy economic burden on low-income countries.

Domestic violence is widespread in the Kyrgyz Republic. There is a scarcity of disaggregated data about DV, however, and much of the evidence is anecdotal or based on estimates (CEDAW, 2015; Childress, 2018). According to the National Statistical Committee of the Kyrgyz Republic (2015), the largest proportion of victims of IPV are women (96% in 2014), and the incidence of IPV has been steadily increasing (by 1.7 times in 2014 as compared to 2010). The government's 2012 Demographic and Health Survey, which provides the most recent comprehensive official data on IPV in Kyrgyzstan, indicates that 23% of all women age 15–49 have experienced physical violence at least once since age 15 and the rate is even higher—28%—among married or formerly married women; in addition, 4% of married or formerly married women reported exposure to sexual violence and 14% reported experiencing emotional abuse inflicted by their current or former partner (National Statistical Committee [NSC], Ministry of Health [MoH] & ICF International, 2013). Of victims experiencing physical or sexual violence of any type, only 39% sought assistance (NSC, MoH and ICF International, 2013). Evidence gathered by organizations working against DV in the Kyrgyz Republic suggests that the actual number of women experiencing DV is much higher than official reports reflect, and that most abused women remain silent because of the widespread belief that domestic violence is a private matter that should not be discussed outside of the home.

### Legal Framework on Domestic Violence and Gender Equality

In response to the international human rights efforts, the Government of Kyrgyzstan has openly acknowledged that DV is a public policy and not just a personal family matter and has adopted both policy and legal measures to address the issue. It ratified the CEDAW Convention in 2002 (CEDAW, 2015) and adopted a Law on Social and Legal Protection from Domestic Violence in 2003 (Law of the Kyrgyz Republic on Social and Legal Protection from DV, 2003). The 2003 DV Law prohibits physical, psychological, and sexual violence (including marital rape) among family members and introduces provisions for restraining orders and other protective measures. Although these steps helped to acknowledge the problem and helped women obtain temporary restraining orders, they also revealed some challenges and inconsistencies in the implementation of the law. The institutional failure to provide supportive services to victims has been widely documented by non-government institutions (Human Rights Watch, 2017) and recent research reports (Childress et al., 2022, 2023).

In 2017, President Almazbek Atambaev signed a new law and accompanying legislation that replaced a 2003 version of the law to include measures to improve protections for victims of DV and strengthen police and judicial response (Human Rights Watch, 2017). This was made possible by new efforts by local organizations and women's rights activists who advocated for and revised the new law. The 2017 law (Law of the Kyrgyz Republic on Protection from DV, April, 2017) increases safety for survivors by limiting access to purchasing or possessing firearms; expanding legal protection for a wider range of harms including psychological, economic, and physical abuse as well as neglect; requiring police to record a DV complaint from anyone, not just the victim; allowing any survivor to be eligible for shelter, medical care, and mental health services regardless of whether criminal proceedings were opened in their case; introducing the behavior modification program for offenders; and outlining the roles and responsibilities of numerous government entities in cases of DV, including local self-governing bodies (Human Rights Watch, 2017; UN Women, 2017).

Although the 2003 and 2017 Laws made important strides toward criminalizing DV, significant gaps in implementation and enforcement remained. A critical lack of services for victims and the ineffectuality of the legal system and law enforcement intervention have created widespread barriers to obtaining support (Childress & Hanusa, 2018). Victims fear social sanctions and worry that reporting will jeopardize their children's futures and lead to retribution from their partners. Within Kyrgyz society, there is broad cultural acceptance of DV in conjunction with a lack of trust in the ability of formal organizations to provide help (Childress, 2018; Childress et al., 2019). Public views on women's rights and gender equality in the country are polarized, and social media posts increasingly portray social movements that promote gender equality as a Western concept imposed on an imagined traditional culture. A significant percentage of the Kyrgyz population has been hesitant to change their views on the roles that are

culturally assigned to women (Muldoone & Casabonne, 2017). A gender inequality index compiled by United Nations Development Programme (2013) shows rising gender inequality in Kyrgyzstan, especially in the rates of labor force participation.

Kyrgyz society's romanticization of a woman's role as a wife whose life is limited to the domestic sphere has normalized discriminatory practices aimed at girls and women (Asian Development Bank, 2019). Women face rigid gender stereotypes that have been embodied in national traditions that normalize subservience to men and can legitimize violence against women. Specifically, the social construction of marriage, the stigmatization of divorce, and cultural norms around the roles of mothers-in-law and daughters-in-law create conditions that legitimate DV (Childress, 2018). Marriage practices (including bride kidnapping), the limited participation of women in public and political life, and the widespread acceptance of patriarchal attitudes have rendered women vulnerable to DV (CEDAW, 2015**)**. 

Prior research has shown that stereotypes influence Kyrgyz women's and men's understanding of violence perpetration (Childress, 2018). Only recently have researchers begun to identify and address the cultural factors that influence the prevalence of DV and create barriers to help-seeking (Childress et al., 2018; 2021). This study represents one of the first efforts to focus on the perspectives of frontline professionals in the health, social work, and legal system to examine how Kyrgyzstan's social and cultural norms influence the prevalence, justification, and perpetuation of DV. Professionals from the criminal justice, public health, and social service sectors were recruited to engage in discussions that shed light on the cultural beliefs and practices that perpetuate violence. 

## Theoretical Foundation

The theoretical foundation underlying the analysis is an ecological approach to DV, which conceptualizes violence as a multifaceted phenomenon based on the interplay among personal, situational, and sociocultural factors (Heise, 1998). A wide literature using this approach has examined the impact of cultural beliefs on DV by identifying a variety of social constructions that serve to justify and perpetuate DV. These constructions include cultural worldviews (for example, individualistic vs. collectivistic), structural systems and notions of womanhood (for example, like patriarchy, masculinity, and the sense of autonomy and self-concept), expectations around gender roles via socialization and training, and the isolation of wives (Campbell, 1992; DeKeseredy, 2021; Kasturirangan & Williams, 2003; Rydstrom, 2003; Walker, 2020; Yoshihama, 2002). Anderson & Umberson (2001), for example, highlight the role of societal constructions of masculinity in the narratives of a diverse group of men participating in batterer intervention education. They demonstrate how the use of social narratives around masculinity and gender, such as upholding men as rational and women as irrational or emotional, contributes to the description and justification of actions by abusers. Kelly (2003) outlines the way that underlying messages found in many societies, suggesting that DV is a private matter to be dealt with inside a family unit, have created barriers for survivors of violence. These messages contribute to isolation and the creation of shame and vulnerability for survivors that further entrench violence within societies. This literature calls for culturally sensitive and feasible approaches to reducing DV that complement the cultural traditions of women from different backgrounds and strengthen women's roles, self-awareness, self-sufficiency, and female coalitions (Fernandez, 2006; Yoshioka & Choi, 2005).

## Methods

This study was part of a larger study conducted to examine barriers to help seeking from DV in low-income contexts and among immigrants and refugee populations in the US. The current study focuses on the cultural barriers mentioned by professional service providers working with abused victims in Kyrgyzstan. Qualitative, grounded theory research methods (Glaser & Strauss, 1967) were chosen for the study for their ability to provide in-depth analysis and to expose layers of nuances between the popular understanding of DV, the sociocultural contexts, and their influences on human behavior.

### Sample and Data Collection

The sample included 83 expert interviews (20 interviews and 8 focus groups) held with professionals in the criminal justice, public health, education, and social welfare sectors (see Table 1. Participant Characteristics). The informants were selected to include individuals from each of these sub-segments and others identified during initial interviewing, based on the theoretical sampling technique (Glaser & Strauss, 1967). The purpose of theoretical sampling is to select episodes and events based on the dimensions and categories that they represent. The researcher seeks subsequent cases and examples as these observations progress based on their potential to strengthen and elaborate newly emergent theoretical constructions (Charmaz, 2006).

**Table 1.** Participant Characteristics.

Sector	#	Male	Female	Ethnicity	Govnt	NGO
Law Enforcement	11	11		Kyrgyz	11	
Judicial Sector	9	1	8	Kyrgyz	9	
Ombudsman	1	1		Kyrgyz	1	
Legal Aid	11	3	8	1 Russian & 10 Kyrgyz	11	
Public Health	9	1	8	Kyrgyz	7	2
Social Services	37	1	36	4 Russian & 33 Kyrgyz	20	17
Education	5		5	Kyrgyz	5	
**Total**	**83**	**18**	**65**	**5 Russian and 78 Kyrgyz**	**64**	**19**

The initial set of criteria to select the first participants included being at least 18 years old, having worked in their current position for at least one year, being able to provide informed consent and follow study procedures, and speaking Russian or Kyrgyz fluently. The initial sample included social service providers who worked in the violence against women crisis response sector. The interviews revealed the need to expand the focus to family problems pertaining to children's exposure to violence and other adversities and to include experts who provide services in the educational, local government, health, and law enforcement sectors. The further comparison revealed that organizations that prioritize violence prevention and family-based preventative actions for children at risk should be included in the data collection. Following the theoretical sampling procedure, in-depth interviews and focus groups resulted in the following groups of professionals: (a) social workers and psychologists from the local government, non-governmental organizations (e.g., crisis centers, shelters, and child and family support centers), and educational establishments; (b) judges, lawyers, and legal advocates from local courts and legal aid clinics, and district police; (c) social pedagogues^2^ from schools and teacher training institutes; and (d) public health workers (doctors and nurses) from the maternity hospitals and primary health clinics.

### Data Analysis

Semi-structured interview guides (in Russian and Kyrgyz) were used to interview the participants (see Appendix 1. Interview Guide). All interviews were audio-recorded, transcribed verbatim, and translated into English. A team of three researchers coded the transcripts using Nvivo12 Qualitative Computer Software. The “open coding” technique was utilized to identify the main themes and categories using a word-by-word examination of the data. “Axial coding” was then used to further reassemble the data fractured during the initial coding procedure. The development of themes and subthemes based on the data was the next phase in the investigation (Charmaz, 2006). Data analysis was strengthened by analytic triangulation, peer-debriefing, and the inclusion of instances of raw data in the findings presentation to assure the reliability and consensus of the conclusions (Lincoln & Guba, 1985). Permission to conduct this study was obtained from the Institutional Review Board of the first author's University in the United States. The study protocol adhered to the World Health Organization guidelines (2001) for conducting research on DV. No names or identifying information were recorded, and pseudonyms are used to protect the anonymity and confidentiality of the study participants.

## Findings

**Figure 1.**
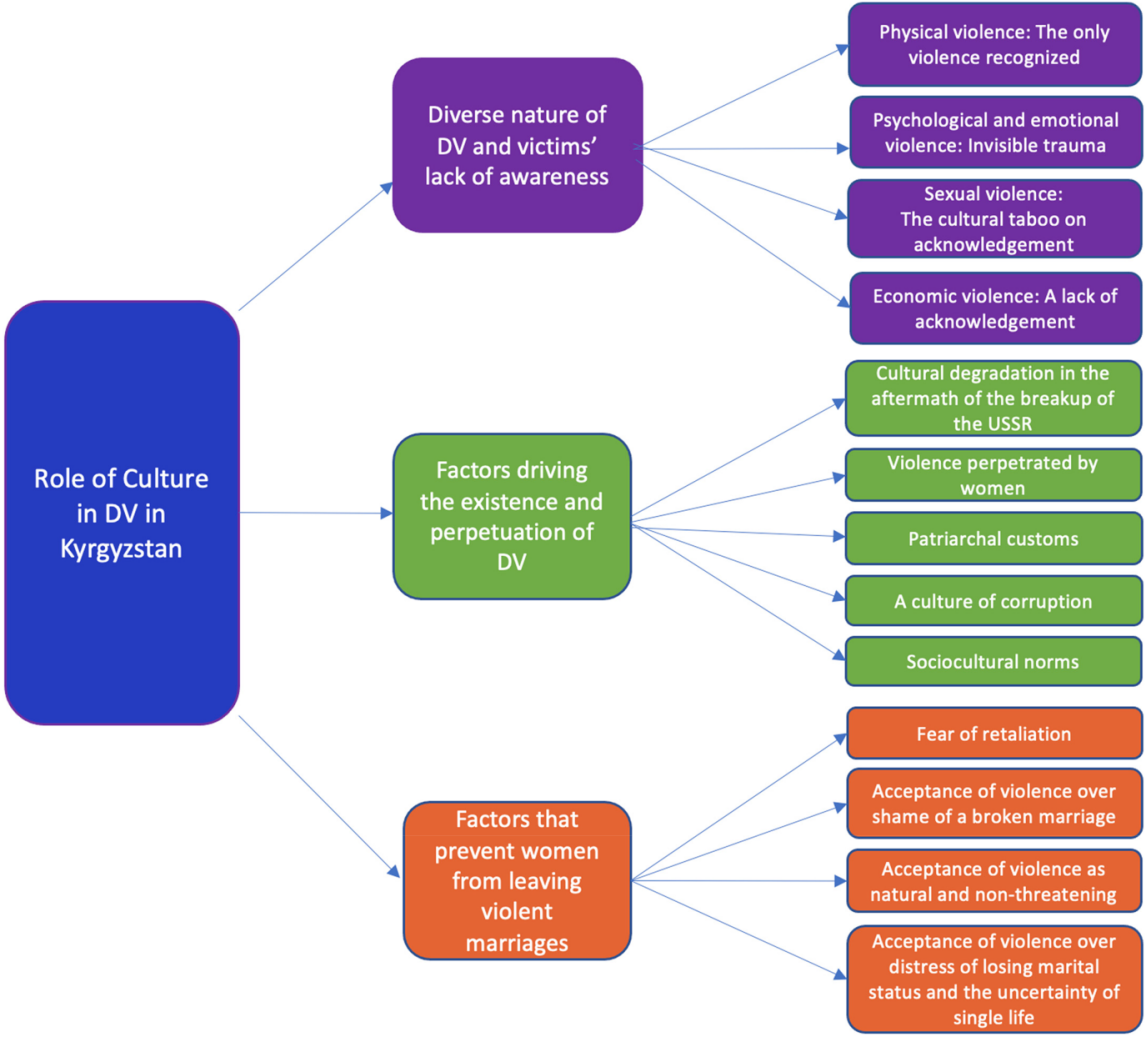
Culture and its effect on violence justification and perpetration in Kyrgyzstan.

Three main themes emerged from the interviews: (a) diverse nature and lack of awareness of DV in Kyrgyzstan; (b) factors driving the existence and perpetuation of DV; and (c) factors preventing women from leaving violent marriages. Each theme is elaborated through discussions of multiple sub-themes (see Figure 1. Culture and its effect on violence justification and perpetration in Kyrgyzstan).

## Theme 1. The Diversity and Lack of Awareness of DV

Respondents characterized DV in Kyrgyzstan as a diverse set of experiences, containing four sub-types: (a) physical violence, which is justified by both perpetrators and victims; (b) psychological and emotional violence, which inflicts invisible trauma; (c) sexual violence, the recognition of which is taboo; and (d) economic violence, which is rarely acknowledged.

### Physical Violence

Study respondents felt that physical abuse was the most pervasive form of DV in Kyrgyzstan. Respondents explained that Kyrgyz culture demands that a man present himself as strong, unemotional, and capable of controlling his family and that disciplining a wife demonstrates these qualities. Most men who use physical force against their wives use religious texts to justify their actions. Using physical violence against an intimate partner is also sometimes viewed as evidence that a man has affection for and feels close to his wife. Kutman, a lawyer with a non-profit organization, explained this societal belief: “He who beats, loves you. You can’t complain or leave; relatives will point fingers at you. According to Sharia,^3^ complaining or leaving is impossible; it is not allowed.” An official from an NGO working on DV issues expressed:

It's slightly offensive when young, progressive girls say, “If your husband beats you, it means he loves you.” This means that people's thinking has not changed yet [from considering physical violence as evidence of intimacy to accepting it as a crime]; society is not ready for such a drastic change.

### Psychological and Emotional Violence: Invisible Trauma

Respondents explained that most women in Kyrgyzstan do not recognize the existence of psychological violence because only extreme physical violence is commonly considered spousal abuse. The low level of education in the country, a general lack of awareness about different types of violence, and limited media coverage of violence prevention programs mean that most Kyrgyzstanis do not recognize psychological violence as abuse. In many cases, neither the abuser nor the victim is aware of the existence of psychological abuse. Baktygul, a supervisor at a children's center, says that only extreme physical violence is recognized as abuse:

In most cases, women do not consider it to be violence when they are humiliated or insulted at home. A husband can insult her, restrict her freedom…They [Kyrgyzstanis] think that violence means only when she is beaten to death and cannot function physically. Or if there is some kind of unlimited sexual violence, let's say.

The professionals agreed that the lack of any physical marks in cases of psychological violence often led victims to underestimate the traumatic effects of the abuse, and society to view the consequences of psychological violence as unimportant. Further, it is culturally accepted and even expected that husbands will control their wives. Saadat, a clinical psychologist, concluded that children accept psychological violence without challenge because they have experienced a patriarchal education. She explained, “Psychological suffering is not generally considered suffering; only physical suffering is taken into account. For example, there is a scar, or a concussion, or fracture, or death. Psychological suffering is greatly underestimated.”

The respondents shared that the expectations of husbands are typically low in Kyrgyz society. If a man is employed, provides for his family, and is neither an alcoholic nor physically violent, he is considered an “ideal” husband whom a wife should have no complaints against. In addition, because parents rarely teach their children about the importance of psychological health, most abusers do not understand the impact of emotional mistreatment. Saadat (clinical psychologist) commented that victims feel abuse but cannot pinpoint how they are being abused, because they lack awareness of psychological violence:

They understand that they are suffering, but they cannot explain the reason. For her, it seems that he doesn’t drink, so why is it so bad? Then you begin to find out and see that there is a lot of emotional violence. In other words, he humiliates and devalues her, does not allow her to see relatives and isolates her from friends, completely controls her life, he is jealous, and so on. And they say, “They don’t beat [me], they make money; I can’t understand why it is so bad.”

Psychological violence can have serious consequences, including depression or anxiety. Inflicting violence through insensitive words and passive aggressive behaviors can distress victims, but because psychological violence does not produce physical injuries, victims cannot provide evidence of abuse. This dilemma prompts some victims to take extreme steps when they cannot take any more abuse. Saadat continues, noting, “This negative psychological pressure leads to depression and probably, to suicide. It is very hard to prove [that it brings women to] death…. It makes her feel bad because of the words of others.”

### Sexual Violence and the Cultural Taboo on Acknowledgment

The professionals reported that there is an acute lack of awareness of sexual violence in Kyrgyzstan. Because cultural norms dictate that a married woman has a duty to please her husband, most Kyrgyzstanis do not believe that sexual violence is possible within marriage. Further, because sex is a taboo subject, there is no formal education on sexual behavior. The culture of silence on the topic of intimacy results in couples relying solely on their personal experiences to gain knowledge about sex or sexual abuse. Ainura, who works at a social service organization, notes that sexual themes are taboo subjects that victims do not reference directly:

Usually they talk about the physical, emotional, and psychological violence, but when it comes to sexual violence, they prefer to keep silent. You understand that it is there, but they usually don’t talk about it. Generally, they talk about physical violence and say, “[He] beat me” without understanding that they have also been emotionally and psychologically abused, their needs have been disregarded.

Aigul, a program manager at a non-profit organization, explained the Kyrgyz concept of shame that victims associate with talking about sexual violence:

Sexual violence is a taboo subject in Kyrgyzstan. Women rarely ask for help when they face it. The root of the problem lies in the lack of families’ sex education, referring to it as “shame.” We have a word, *uyat*, shame. Mothers do not provide sex education to their daughters who are soon to-be-brides, especially in rural areas, and it [sexual violence] is considered normal.

### Economic Violence: A Lack of Awareness

Respondents reported that, just like psychological violence, there is little awareness of economic violence, and it is not regarded as violence. Begimai, director of a non-profit organization that provides support for self-help groups and small businesses described women's surprise upon learning about economic violence, “When we held meetings and discussed the types of violence, they were shocked, ‘I cannot believe that there is economic violence. It turns out my whole life I have been subjected to this type of violence.’”

The professionals also emphasized that Kyrgyzstan's patriarchal culture poses a challenge to women's independence. Most girls are raised to become “good wives,” which typically means prioritizing household activities, even over their education. Once married, women become financially dependent on their husbands without right to paternal property. If a woman wants to study or work, she must get approval from her husband. A woman's liberation, including her financial independence, is contingent on her husband's consent. Begimai described the potential consequences of this dependence: “In the end, if a family gets divorced, then the woman is left with nothing…. She lives in the family for several years, and in the end she does not have the right to any property.” Respondents explained that in Kyrgyzstan houses are often registered in the name of the parents-in-law to prevent the loss of property to a daughter-in-law in a divorce. This reflects women's position at the bottom of the family hierarchy. Aigul, a program manager at a social service organization, said, “Men give ownership of their [homes] to their mothers or fathers. For cases where something bad happens—to make sure that a daughter-in-law gets nothing.”

As the respondents explained, Kyrgyzstan is a patrilocal culture in which women live with their husband's extended family after marriage. Cultural norms dictate that married women prioritize the needs of their in-laws’ over their own. Considerable time and effort go toward managing the household and catering to the in-laws, and women who work outside the home cannot devote as much time to these duties. In these cases, a husband can refuse to grant his wife permission to join the labor force, making his wife dependent on him financially and otherwise. Baktygul, a supervisor at a children's non-profit, recalls how in-laws prevented her from working, “My father-in-law and mother-in-law didn’t allow me to work. At the end I got a job…but there were fights.”

## Theme 2: Factors Driving the Existence and Perpetuation of Domestic Violence Cases

Participants cited five main factors driving the perpetuation of DV: (a) cultural degradation in the aftermath of the breakup of the USSR; (b) violence perpetrated by women; (c) patriarchal customs; (d) a culture of corruption; and (e) sociocultural norms.

### Cultural Degradation in the Aftermath of the Breakup of the USSR

Participants expressed concern that cultural degradation in the aftermath of the breakup of the Soviet Union was contributing to DV. For example, Gulira, a foster mother working in the government, believed that the disappearance of previous value systems was responsible for the rise in violence in Kyrgyz society, expressing hope that old value systems would not change: “As the Soviet Union collapsed, our mentality has changed. The previous Kyrgyz values are disappearing. The mentality of people transitioned to capitalism and changed. It would have been nice if it did not change.” Kyrgyzstanis often romanticize the past as a morally superior time when couples stayed true to their marriages despite challenges and refrained from divorcing over petty issues. Respondents felt nostalgic about earlier eras and stated that during that time *aksakals^4^* (local elders) were able to effectively resolve DV cases in rural areas, whereas now such cases are handled by the police or present-day *aksakals*, whom the respondents characterized as corrupted by capitalism. Sairagul, a professor of psychology and social work at a local university, lamented the changes that came with the end of socialism:

How much has the market economy destroyed our traditional values today? Even the institution of *aksakals* has degraded. Previously, *aksakals* were considered a source of fair and unbiased judgement. Now, if an *aksakal* has a son who is richer and more successful than others, he will bless and care only for the rich son…. In the past, *aksakals* spoke only the truth and taught not to take away someone else's property.

Respondents asserted that because the breakup of the Soviet Union had dismantled the traditional system, women who had previously served their husbands and in-laws now refuse to do so. They blamed the high divorce rate on the new culture's devaluation of marriage, and women looking for an easy escape from responsibilities. Klara, a social worker active in the local government, lamented, “Women used to make a marriage work. Nowadays, they do not [want to] serve their husbands and in-laws. They just fight, divorce quickly, and… want child support. I was in court yesterday participating in seven trials of divorces. Seven!” Participants felt that the imposition of European laws in Kyrgyz society was hurting the family system because these laws were incompatible with the social and cultural fabric of Kyrgyzstan, claiming that the Western concept of gender equality had encouraged women to attack men and see them as vulnerable. Cholponai, a psychology professor, shared:

The families have evolved: if previously women could not raise their heads, now they are attacking men. No one protects men; it is a bilateral violence then. It seems to me that too much attention and effort are given to the question of gender equality. What is gender equality?

### Violence Perpetrated by Women

Respondents considered the psychological violence that mothers-in-law and sisters-in-law inflicted on married women to be more damaging to a marriage than that inflicted by a husband. They believed that women's lack of empathy toward another woman in a challenging situation drives DV. Sairagul, who teaches psychology, asserted that most violence against women emanates from the women in the husband's family, “There's also violence against women perpetrated by women. For example, a husband's little sister, although she may encounter the same situation herself when she gets married, she still forces her parents and brother to give their daughter-in-law a certain role.”

Respondents tended to hold women in the husband's family responsible for much of the violence perpetrated against daughters-in-law. They blamed bride kidnapping on mothers-in-law, claimed that sisters-in-law often provoke their brothers by emphasizing his wife's poor housekeeping skills, and said that the women in families often settle personal grudges by encouraging violence. Sairagul described multiple types of this violence:

In our tradition, it is not men who first commit violence, but women. Let's take an example of *ala kachuu* [bride kidnapping]. Who convinces a bride to stay and agree to marry? The women will claim, “I was also kidnapped and agreed to marry; it is okay if you do the same.” None of the women takes into account the opinion of a [kidnapped] girl, and this violence subsequently continues. There are frictions and expectations from the mother-in-law that a *kelin* [a newly married woman who recently moved into her husband's family home] must serve as a slave: get up early in the morning [to clean or serve breakfast] … This also relates to the sisters of the husband. They are the first to complain, “Your wife is not doing housework properly. It is our fault that we gave her too much freedom.” Female relatives of the husband push him to commit violence against his wife…out of jealousy or grudges.

Participants said that married women are more fearful of their in-laws than their husbands, describing how men use physical violence to show that they are in control. Kanymgul, a professor of psychology, shares:

The root of the problems is not in men but in female relatives who push men to violence. Female relatives advise a man to kidnap the girl they [the women] like most, but not the girl the man likes. Then men begin to drink alcohol, beat their wives, and…get divorced. One of my clients told me that she was more afraid of her mother-in-law than her husband. Her husband was inclined to let little things slide, but his mother would encourage him to beat her for burning the food. He was always more aggressive toward her when the mother-in-law was around.

The interviews also revealed that when a couple separates, it is blamed more on the women in the family and less on the husband. The respondents reported that the interference of the husbands’ family causes conflicts between partners. Cholponai, a professor, explained:

They [women in the family] claim, “*Kelin* has to live through these things, too.” Therefore, divorces happen because of [female relatives]… Often, it is easier for women to find common ground with her husband rather than with the women in his family. … Why not leave a young family alone and allow them to live independently?

Cholponai also believed that the physical and sexual violence men unleash on their wives is a reaction to psychological violence, including badgering and complaining, that wives inflict on their husbands. Questioning a husband's ability to look after his family's needs (e.g., pointing out his insufficient earnings) is considered unjustified “psychological violence.” The respondents implied that women often trigger violent reactions by aggravating their husbands; arguing that women should be held responsible when men react to such provocations with violence. Cholponai described violence as a two-way street with women initiating the cycle by inflicting psychological violence on men, and men, in turn, inflicting physical violence on women:

There are situations when a woman often bothers her husband by complaining, “You do not work! When will you make money?” and for him, it will be easier to hit her. In the end, he will be an abuser, but in fact, she is the first to start psychological abuse [over him]. After a day or two, he drinks alcohol with friends, returns home, and the story of violence repeats again. It is [unfair] to say here that only men commit violence.

Both psychology professors believed that the women in a husband's family inflicted violence on married women. They viewed husbands as a channel for delivering this violence and felt that the women in families were the true perpetrators, provoking husbands to lash out when they believed a woman was not being a dutiful daughter-in-law or not living up to expectations.

### Patriarchal Customs

Participants attributed violence to the custom of a bride moving into her husband's house after marriage. This tradition places brides in a family system with their husbands’ family members, relegating them to a low status and increasing vulnerability to abuse. In-laws, especially mothers-in-law, who are powerful figures in Kyrgyz culture, exert a strong influence on newly married couples (Childress, 2018). Family members expect daughters-in-law to fulfill the household duties and cater to the wishes of the family. Sairagul, a psychology professor, explained, “After marriage a young girl comes to a family where everything is controlled by her husband's parents, and she lives in a slavish condition.” Even if the husband is absent, his wife is expected to take care of his extended family. Women often want to join their husbands but family members forbid this and engage in psychological violence. Sairagul continued:

Many women want to migrate with their husbands to Russia, but husbands often leave them at home. [These women] often write, “I am not on good terms with my mother-in-law. I want to save my family and live with him. I do not know why my in-laws do not let me go to him.” However, these women live without [their] husbands for years and suffer harassment from relatives.

Participants spoke about the *kelin* system, in which a wife's movements are limited, and her life is managed by her mother-in-law. Few women ever break out of this system. Respondents reported that patriarchal customs such as living in a husband's house after marriage, wives taking care of a husband's family when he migrates for work and forcing young couples to follow the customs of the husband's household encourage violence against married women.

### A Culture of Corruption

The respondents agreed that one reason DV persists is because there is little trust between abused women and formal services such as police. Because women often retract registered complaints against perpetrators and reconcile with them, the police do not take complaints seriously. Women who want to pursue a case are hindered by the weak implementation of DV laws—the police often “close the case in the Kyrgyz manner” in collaboration with the perpetrator. Thus, the trust deficit between victims and the authorities creates a vicious cycle: women have no faith in a system that closes registered cases, forcing them to reconcile with their abusers. Aigerim, a social worker at a children's organization, offers an example of this cycle, “The woman writes a statement, turns to district police. A policeman checks in for 2–3 days and then leaves [for good]. They close the case, and the violence continues. There is no preventative work.”

Police often do not explain the punitive measures for DV. Uson, who works at a children's organization, says that the police dismiss cases of DV because they believe the family will reconcile, and it is not worth investing the effort. He concludes,

The family turns to the police and files the complaint, but our law enforcement are accustomed to the fact that the family can write the statement and then withdraw it after three days. … They want the family to make a decision and continue to live together. But they do not carry out preventive explanatory work. They do not say that it is punishable. … They think, “Let the family fight today, because after 2–3 days they will reconcile.”

There are several possible reasons victims choose to move back with their abuser including the stigma associated with divorced women, economic dependency on the abuser, and fear of the unknown. Myskal, a social worker at a children's non-profit, says it is frustrating to obtain a protection order only to have the victim reconcile with the abuser. She recounts:

She and I obtained a protection order. … We were standing up for her rights, but she decided to reconcile. She saw her husband and immediately reconciled with him. … Of course, psychological factors also play a role. Families fall into the victim-rescuer-persecutor triangle. They have been living with it [violence] all their lives … and it is very difficult to break this cycle.

### Sociocultural Norms

Respondents reported that victims fear stigmatization by relatives and neighbors for reporting a DV incident to the police. According to Kyrgyz sociocultural norms, family affairs, including DV, should be kept within the private sphere of the home. When wives involve the police and the issue becomes public, community members perceive the victim as violating an unspoken rule of society and penalize her for reporting. Erkeayim, who was associated with a children's non-profit, described the trauma of reporting DV to police:

She turned to the police department. But relatives, neighbors will remember it all their lives, they’ll come up with some jokes or with a nickname. It is a psychological pressure. Imagine she is celebrating some event having 20 people around and someone remembers it. …Then you think to yourself, “Why did I do it?”

When a woman makes a public disclosure of private affairs, her relatives view her actions as bringing shame to the family and will remember them for a long time. Victims may be taunted for their choice for years, leading them to regret going to police. These repercussions discourage other victims from reporting. Erkeayim continued, noting that children who witness these insults experience intergenerational trauma and may prevent their mothers from registering a DV case because they fear reprisals. She explained, “This is traumatic for children … Sometimes children stop their parents from filing a complaint.”

Respondents observed that in Kyrgyzstan (as in any patriarchal society) the concept of an honorable woman is based on her status as a married woman. Women who separate from their partners are perceived as having a suspect character. Saadat, a clinical psychologist, said that divorced women are despised, and the treatment they receive from relatives is so discriminatory that they often prefer to reconcile with the abuser. She concluded,

Even people with higher education say that the woman will lose respect. There were cases in which a woman said, “I got divorced for a while and started to notice that when I came to [my parents’ house] along with my mother-in-law and husband, they used to place me in a respected spot at the table. After I got divorced, my relatives began to [give those places] to my younger sister.” There is stigma even from the relatives who, in general, should be supportive.

The shame that befalls a divorced woman and her family is so formidable that most women would rather endure abuse than divorce. A divorced woman will almost certainly receive no support, even from her immediate family members, who will blame her for bringing shame to the family. Bektur, a police officer, says that the onus is on women to maintain the family system, and any woman who breaks this sacred tradition is considered an outcast, “If she goes to her parents’ house, they will scold her by saying, ‘Are you going to bring shame to our family again? Go back to your husband.’”

DV is normalized and is considered a normal part of a married life in Kyrgyzstan. The widespread acceptance of DV across social classes allows husbands and family members to justify violence. Ainagul, a worker at a maternity hospital, says that the normalization of violence is the reason many women believe that abuse is simply part of married life; she observed, “Most of them do not understand what is happening and do not recognize that they are living with violence.” Kyrgyz women experience social conditioning from childhood leaving them with low self-esteem and a compromised sense of agency. Darya, the coordinator of a crisis center, feels that abused women do not value themselves to believe they deserve a violence-free life:

When you start counseling them, they start talking about their husbands and how they beat them. You ask them some questions about themselves: “Well, and you?” but they again go, “Well, my husband…” These are people with low self-esteem, [a lot of] self-doubt, who never thought for and loved themselves.

The dominance of a patriarchal culture that reduces a woman's worth to her marital status and how well she can sustain the family is one reason Kyrgyz women endure DV. The ostracism that divorced women experience deters many from pursuing their cases. Many victims choose to lead an “honorable” but violent life rather than seeking safety and dishonor.

## Theme 3: Factors That Prevent Women From Leaving Violent Marriages

Participants discussed six main factors that prevent abused women from leaving violent marriages: (a) fear of retaliation, (b) acceptance of violence over shame of a broken marriage, (c) acceptance of violence as natural and non-threatening, and (d) acceptance of violence over distress of losing marital status and the uncertainty of single life. These sub-themes are explained in the following sections.

### Fear of Retaliation

According to the participants, a culture of silence is pervasive in Kyrgyz society, whereby wives do not discuss their marriages or violence for fear of retaliation. Women fear physical abuse as punishment for talking about violence, including, in extreme cases, murder. Sveta, a lawyer at a legal aid clinic, explained the casual manner in which authorities handle violent retaliation by abusive husbands. Even cases of attempted murder are downgraded to less serious crimes despite severe physical harm.

A husband tried to push his wife out of the car because they had some type of disagreement… She was unconscious for 3–4 h. She had very deep wounds that required stitches. … A [forensic] examination was conducted and was [categorized] as a less serious [crime], although she was in hospital for 15 days.

### Acceptance of Violence Over Shame of a Broken Marriage

In the Kyrgyz tradition, a married woman does not have the right to return to her parents’ house and doing so is considered shameful. A broken marriage brings dishonor to the maternal family, and thus parents would prefer that a married daughter stays in a violent marriage rather than return to live in the maternal home after a separation or divorce. Family members and the community blame the woman for not enduring violence, for provoking her husband, and for not being able to keep the family together. Most Kyrgyzstanis accept violence as a normal part of marriage and view a woman's refusal to endure violence quietly as a weakness. Baktygul, a supervisor at a children's non-profit organization, recalls a situation where a mother discouraged her daughter from separation because of the shame associated with a broken marriage. Baktygul says the following about the conversation:

It was in our practice—a woman lived in a family and her husband was a monster. He used to beat the woman to bruises every day. The woman asked her parents for help and in response they said, “Well, all husbands are like that; what did you expect when you got married?” [Her] mother said, “I lived like this. Do you think your father did not beat me? So, I am 70 years old and just a couple of years ago he stopped beating me. You know it yourself, and it is our tradition. It is normal, and you have to accept it. And what are you going to do with five children? If you get divorced, where will you go? We won’t accept you with children. How are you going to survive?”

Salkynai, a representative of a Social Protection Department, also reported that parents expect their daughters to maintain a family, and explained that if they fail to do so, parents blame their daughters for bringing shame on the family.

When I was fighting with my husband a lot, my father told me, “I am ashamed of you.” I stayed and lived there because of these words. I was afraid of my father; [I thought,] “My father will scold me. No, I will not leave.”

### Acceptance of Violence as Natural and Non-Threatening

Respondents shared that violence is pervasive and is widely accepted as a natural part of marriage. Children grow up observing violence in the family and internalize it as a normal event that occurs in every family. As adults, women tolerate violence because their mothers and female relatives have done so, and men inflict violence because they have grown up watching their fathers and male relatives do the same. The culture of violence is accepted by both men and women, and very few Kyrgyzstanis view DV as improper, much less a crime. A program manager from the social services sector observes, “Women themselves?! They accept it. [Violence] continues while she endures everything, giving birth to five or six children. She thinks that this is all normal.” Oksana, a psychotherapist at a crisis center, says the problem starts when women are taught that they bring violence on themselves through their own actions. She explains:

In many families, parents or relatives of a woman tell her that she should endure it; that it's all women's destiny; that she should be wiser, cunning, patient; that all women suffer, and that she should [too]. And generally, if the husband offends, humiliates, or beats you, [it] means that it is also her fault.

Darya, a coordinator at a crisis center, discussed the pervasive culture of secrecy surrounding DV, noting that talking about it would bring shame to the family, “Many families hide it because it is *uyat* [shame]. They think, ‘Let her suffer so no one would know. How will we look in the eyes of the neighbors?’ This is just a very frequent factor.”

### Acceptance of Violence Over Distress of Losing Marital Status and the Uncertainty of Single Life

Participants discussed how a married woman in Kyrgyz society has a status but loses this status if she gets divorced. A woman without the protection of a husband is viewed with suspicion and disrespect. Thus, women are often advised to endure violence rather than get divorced or separated. Baktygul, a supervisor at a children's center, explained, “They say, ‘Who will you be [without a husband]?’ We still have a concept of being an incomplete member of society if a person does not have a family of her own.” Margarita, who works as a lawyer, said it was difficult to convince women to separate from their abusive spouses. When she suggested separation, victims often responded that violence has been occurring for generations and it would be a shame for them to break up a marriage because of it now.

The respondents shared that a woman with no social support system is likely to return to her abuser because she could face homelessness and economic uncertainty. Mairam, an official at a shelter that provides short-term residential support to abused women, said that without transit centers, women could fall into destitution:

For example, a girl has no one, no family or relatives. During this time [residence in the shelter], they only come to their senses, but are forced to return to the same situation. In order to bring stability to life, they need more time so that they can go through integration and rehabilitation. In this regard, we plan to open transitional housing, where women can stay for three years and work.

Edita, a social worker at a shelter, also observed that many victims return to their abusive husbands, because they have few options beyond marriage. Edita summarized, they do not have parents to go to, a mother and a father died, or a mother got re-married and cannot take care or provide help. In most cases, [victims] have no university degrees, no profession, and they cannot sustain themselves, let alone their children. Thus, they return to their husbands.

Margarita came to a similar conclusion, noting that the women who return to their abusive husbands are generally those with no social support system. She offered the following case as an example, “She returned [to her abusive husband] out of hopelessness; she had nowhere to go. Her mother lived with [her] half-sisters from different husbands…She left her husband three or four times. He beat her, but she returned.”

Most victims have no financial backing or independent income source to support themselves and their children if they divorce. As described above, parents-in-law often register the family's house on their own names, so victims cannot lay claim to it. Baktygul described her own experience with this tactic when she “decided to just run away from the family, where there was no longer a life.” She recounted, “We had a big house. We constructed it for several years, made renovations. When I decided to leave, my mother-in-law reissued the title to ensure that if I filed for divorce, I would have no [property] rights.” Aigul, a program manager at a non-profit organization, also described this uncertainty, “Women often make arguments, ‘How will I feed the children? They will grow up without a father. How will I leave all the acquired property?’ because they leave without anything.”

## Discussion

The narratives of professionals in the study, including social workers, lawyers, educators, public health, and criminal justice professionals, who work directly with victims, offer a deeper understanding of the complexities of cultural beliefs and social norms and their impact on decision-making about DV in the context of Kyrgyzstan. The participants reported that culture has a powerful role in the beliefs and emotions of abused women, leading them to accept and internalize violence as a natural part of married life. Among both victims and members of the public, only extreme physical violence is recognized as DV—there is a lack of awareness of psychological, sexual, and economic violence. Respondents highlighted the perceived degradation of Kyrgyz culture after the breakup of the USSR, women-on-women violence, patriarchal influences on sociocultural practices, corruption in the police force, a lack of faith in the judicial system, and self-blame as reasons for the increase in DV cases. They believed that broad acceptance of violence in intimate relationships and the stigma associated with marital separation was driving a cycle of violence and re-victimization.

While prior studies have documented the influence of culture on DV (Childress, 2018), this is the first study in Kyrgyzstan to focus on the narratives of the professionals who work with abuse victims daily. The results reveal the multiple aspects of culture that affect victims of DV from the perspective of experts who have decades of experience assisting abused victims. Further, the results show that while professionals in the field recognize, at least partially, how Kyrgyz culture facilitates the perpetuation of DV, they feel helpless to change long-standing societal beliefs. The study provides further insight into the cultural complexities of DV within Kyrgyz society specifically and highlights the need for contextualized approaches to understanding DV in Kyrgyzstan.

The experiences of service providers align with the findings of previous studies on the role of culture in DV. Prior research in other countries has identified several cultural factors that perpetuate DV. For example, a study from Bangladesh highlighted the internalization of violence (Schuler & Islam, 2008). Work in Nigeria focused on a culture of silence, economic dependence, and a gender-insensitive justice system (Abayomi & Olabode, 2013). Research from Karachi, Pakistan pointed to the importance of family support from the maternal home, a culture of violence acceptance, and a lack of legal support (Rabbani et al., 2008). Finally, a study of immigrant women in the United States stressed conformity to traditional gender roles (Raj & Silverman, 2002). The findings of the current study corroborate these and other earlier results showing that traditional culture plays a significant role in DV, and that fully understanding the local culture is important for ground-level victim advocacy.

The study shows that victims face pressure from multiple sources such as demands from maternal families to reconcile, a lack of cooperation from police, derision from relatives and neighbors for making domestic issues public, and an economic environment that compels victims to return to abusive relationships. Victims who initially expressed resolve to end their violent marriages often relented when the sociocultural environment created a growing pressure to “save” their marriages for the sake of family or children. As in most patriarchal societies, the norms of Kyrgyz society decree that only women who are married are honorable. Any woman breaking this social norm faces stigma and the withholding of support (Childress, 2018). The study also shows that despite these formidable sociocultural obstacles, many victims find the strength to reach out for help (Childress et al., 2021).

The descriptions of service providers’ experiences can be understood within an ecological framework in which environmental demands and resources help or hinder survivors’ coping behavior and decision-making (Heise, 1998). The ecological model incorporates a survivor's individual situation, her household context, her social environment, and the cultural value system surrounding her. Examining these multiple contexts can help researchers understand why (a) women feel helpless when abuse has occurred, (b) most victims are reluctant to report abuse, (c) abused women withdraw registered complaints and reconcile with their abusers, and (d) how these actions affect the cycle of violence. Based on real lived experiences, this framework surmises the individual, the family, and the cultural environment that reinforces patriarchal existence. These experiences reveal a cultural ecosystem influenced by the norms of post-Soviet Kyrgyz society with strong social and cultural deterrents that impede a victim's ability to escape abuse.

At the *individual level*, victims’ personal characteristics, such as the internalized belief that physical violence is a manifestation of spousal affection, lack of awareness about psychological and economic violence, and the perception that sexual violence is a right of married men, affect their understanding of DV. These individual-level understandings are embedded in broader cultural beliefs—for example, about women's identities and status, gender role segregation, and the property rights—that affect responses to abuse. Intergenerational transmission of cultural beliefs ensures that girls are indoctrinated into social norms at a young age. Changing these deep-seated beliefs may require simultaneous interventions on multiple fronts aimed at both men and women. Interventions targeted at women should seek to condition young girls and women to recognize various forms of violence, speak out against it, and reach out for help. Interventions targeted at men should teach them about gender equality, sensitize them to the importance of healthy relationships, and enforce laws against DV.

At the *family level*, the findings reveal a disturbing pattern of women-on-women violence wherein women family members provoked husbands to inflict violence on their wives to settle a grudge or dispute. The patrilocal family system in which a new bride is expected to live with and take care of her husband's family provides leverage to husbands, who have family support. When a bride does not live up to the expectation of her husband or his family members, she is often subjected to DV. Patrilocality has been associated with increased likelihood of DV for women in other studies (Khalil & Mookherjee, 2019; Turaeva & Becker, 2022). An effect of patrilocality and patriarchal culture is that the husband is expected to side with the parents and other family members when there is a conflict between the wife and the husband's family members. This was the case in a study in Nepal (Nwokolo et al., 2020) where patriarchy is strongly entrenched in the social system. It is likely that Kyrgyzstan being a patriarchal country, there are similar expectations from men where reverence towards family members are concerned.

A study in the Central Asian country of Tajikistan (Turaeva & Becker, 2022) found that daughters-in-law who were living in the husband's natal household were 3.6 times more likely to face emotional violence than married women living in nuclear households. The study found that the high level of emotional abuse happened regardless of the presence of the father-in-law, leading to the conclusion that the responsibility for the violence credibly lay with the mother-in-law. Agarwal (1997) notes that in South Asian countries, mothers-in-law and daughters have stronger bargaining powers than daughters-in-law because social norms allow older women and daughters to be more assertive than daughters-in-law who are expected to be submissive to the husband and his family members. The higher power and rights conferred on the husband and his family members and the likelihood of the husband's female family members exercising the power to control the daughter-in-law could be a reason why Kyrgyz victim was likely to hold the female family members responsible for the violence inflicted on her by the husband.

At the *community level*, the findings reveal little support for victims—in many cases even from professionals working in the field of DV—who report the abuse or leave an abusive relationship. A divorced woman is strongly stigmatized and discriminated against at every level, even by members of her own family. Neighbors and relatives deride victims when they report incidents of DV. The police do not seriously pursue cases because they believe women will reconcile with their spouses eventually, and thus it would be a waste to invest effort in the case. Many police officers perceive victims as deviating from their expected roles when they expose private family matters in public. In addition, if a husband has a friendly relationship with the police, they are more likely to close the case, leaving the victim with little choice but to return to the perpetrator. Thus, victims of DV fail to obtain justice, and the corruption of the police force perpetuates the cycle of violence.

At the *societal level*, deeply ingrained cultural beliefs justifying violence and multiple barriers to help-seeking make it extremely challenging for victims to leave an abusive relationship. Women are blamed for violence and are unlikely to receive support from their birth families. The intergenerational trauma of growing up in an abusive environment leaves many women with the belief that violence is a natural consequence of marriage. Unregistered marriages, a lack of property rights for women, and limited or no sources of independent income mean that women are at the mercy of institutions for support. Future work should aim to identify additional effective routes for culturally appropriate support of victims and service providers who seek to support them, as well as build culturally appropriate integrated prevention programs focusing on strengthening family processes (McKay et al., 1995), co-parenting and father involvement (Cowan et al., 2005), and economic empowerment to improve family relationships and promote family well-being (Ssewamala et al., 2022). These types of programs could be directly incorporated into school curricula and target young men and women as a prevention strategy to expose them to new norms and mental models through structured conversations and peer groups.

Ingrained cultural norms do not shift overnight; rather, such transitions are part of a long process in which every stakeholder from the community, including social workers, police, legal entities, and policymakers, must combine their efforts to increase the awareness of gender equality and sensitization among both men and women. While almost all current interventions target women to provide legal and material support after they experience DV, there must also be a strong commitment from agencies to target men for appropriate interventions. Professionals should receive training at regular intervals so they can reassess their perceptions of gender and apply new conceptualizations in their practice. Such efforts are virtually non-existent in present-day Kyrgyzstan and thus require urgent attention. Institutional support will matter little to a victim if society continues to castigate her and discriminate against her because of cultural norms.

## Conclusion

This study is among the first to shed light on the complexities of cultural beliefs and norms related to DV in Kyrgyzstan. Professionals who work with victims in a number of roles highlighted the powerful role of culture and belief in the experiences and decisions of victims, and the link between broad cultural acceptance of violence in marriage and the ongoing cycle of violence and re-victimization. Scholars, policymakers, and service providers must collaborate to actively dispel widely accepted beliefs about gender, marriage, and women's status, and to break the cycle of abuse providing help at all levels of social ecology.

## Notes

1. IPV falls in the wider category of domestic violence, which refers to the various forms of violence involving members of the same household (e.g., partners, in-laws, siblings, parents, and children) (Childress, 2018). In national contexts where there is little information on IPV specifically, a review of the data on domestic violence can be informative. This is the case for the Kyrgyz Republic. In this study, the Kyrgyz and Russian term translated as “domestic violence” was used almost exclusively; however, the terms used by the authors were retained when citing their individual studies.

2. *Social pedagogy* is a specialization that focuses on the processes of socialization, social education, social protection, and socio-pedagogical support for the child and their social environment. A *social pedagogue* works for an educational establishment (i.e., a secondary school). Their primary function is to organize social protection, education, and the personality development of the student through forging relationships between the family and the school. The target group for a social pedagogue's work is children “in difficult life situations” (e.g., family violence, poverty, orphanhood, homelessness, disability, and exposure to trauma and other adversity) (Law on Social Protection of the Population, 2001; Ministry of Education and Science, n.d.; Regulations for Identifying Children and Families in Difficult Life Situations, 2015).

3. *Sharia* is a body of religious law that forms part of the Islamic tradition and provides for rights and responsibilities between men and women in marriage, divorce, clothing, legal status or education, inheritance, and government laws (BBC, 2021)

4. In some Kyrgyzstani communities, *Aksakals’* (or elders’) courts are used to settle conflicts at the local level. According to the Law on Social and Legal Protection from Domestic Violence (2003), a victim of DV has the right to present her own case to the elders’ court, with the intention of “a public rebuke of the aggressor” (Article 6). According to the law that governs *Aksakals’* courts, the elders have the authority to consider cases involving family disputes, including issues of “marital and family relations, including family violence” (Law on Aksakal Courts, 2002, Article 15). These courts can only inflict sanctions that involve public censures or monetary or material reparation (e.g., warnings and public reproaches, demands for a public apology or material compensation to the victim, or fine up to three times the minimum monthly income) to resolve family disputes (Law on Aksakal Courts, Article 28).

## Appendix. Key Informant Interview Guides.

I. General questions

1) Please tell us what “family violence” means in your opinion? (Can you give examples of cases that you have encountered in your work that illustrate the types of behavior or actions that you consider to be violence in the family. Can you describe the typical cases?).

2) Please tell us about your professional experience working with women affected by DV?

3) Why do you think violence happens in the family? What do you believe are the reasons for DV?

In the following questions, we would like to explore the role of social/health workers in helping women victims of DV.

1) Can you tell me about your role and your organization?

a. What types of services you provide? How do women learn about your program?

b. What services do they typically require?

2) What types of abuse are experienced by women in this community?

a. What are the factors contributing to abuse? What are the major challenges faced by the victims?

b. Where do they seek help? What strategies do women use to cope with violence?

3) What obstacles do you experience in providing effective assistance to women experiencing violence?

a. What shortfalls from the government/NGO have you observed in assisting survivors?

b. Can you explain some of the best practices followed by your organization to assist the survivors? What are some of the best practices that you have observed in other organizations that could be widely applied?

4) What short-term and long-term interventions would you suggest to improve the situation for victims? What can victims, civil society, the government do?

a. Do you have any recommendations for practitioners, researchers and decision-makers in the field of DV?

II. Concluding questions.

1) Do you have anything to add that was not discussed during the interview or anything important that occurred to you during the conversation?

2) Is there anything else that you think we should know to better understand your experience with solving domestic violence problems? Do you have any questions for me?

**III. Debriefing** (a short survey after the interview to obtain information about the interview conducted for the purpose of its analysis and suggestions for subsequent interviews).

## References

[bibr1-10778012231186814] Abayomi A. A. Olabode K. T. (2013). Domestic violence and death: Women as endangered gender in Nigeria. American Journal of Sociological Research, 3(3), 53–60. 10.5923/j.sociology.20130303.01

[bibr2-10778012231186814] Agarwal B. (1997). Bargaining and gender relations: Within and beyond the household. Feminist Economics, 3(1), 1–51. 10.1080/135457097338799

[bibr3-10778012231186814] Anderson K. L. Umberson D. (2001). Gendering violence: Masculinity and power in men's accounts of domestic violence. Gender & Society, 15(3), 358–380. 10.1177/089124301015003003

[bibr4-10778012231186814] Asian Development Bank. (2019). Kyrgyz Republic country gender assessment. https://www.adb.org/sites/default/files/institutional-document/546966/kyrgyz-republic-country-gender-assessment-2019.pdf.

[bibr5-10778012231186814] BBC. (August 19, 2021). *What is Sharia law? What does it mean for women in Afghanistan?* https://www.bbc.com/news/world-27307249 .

[bibr6-10778012231186814] Campbell J. (2002). Health consequences of intimate partner violence. Lancet, 359(9314), 1331–1336. 10.1016/S0140-6736(02)08336-8 11965295

[bibr7-10778012231186814] Campbell J. C. (1992). Wife-battering: Cultural contexts versus western social sciences. In Counts D. A. Brown J. K. Campbell J. C. (Eds.), Sanctions and sanctuary: Cultural perspectives on the beating of wives (pp. 229–250). Westview Press.

[bibr8-10778012231186814] CEDAW. (2015). *Concluding observations on the fourth periodic report of Kyrgyzstan*. https://www.ohchr.org/en/countries/enacaregion/pages/kgindex.aspx.

[bibr9-10778012231186814] Centers for Disease Control and Prevention. (2021). *Intimate partner violence*. https://www.cdc.gov/violenceprevention/intimatepartnerviolence/index.html.

[bibr10-10778012231186814] Charmaz K. (2006). Constructing grounded theory: A practical guide through qualitative analysis. Sage Publications.

[bibr11-10778012231186814] Childress S. (2018). Plates and dishes smash; married couples clash”: Cultural and social barriers to help-seeking among women domestic violence survivors in Kyrgyzstan. Violence Against Women, 24(7), 775–797. 10.1177/1077801217722239 29332501

[bibr12-10778012231186814] Childress S. Aparicio L. Messing J. (2019). Domestic violence in Kyrgyzstan: Finding a voice of strength and empowerment. In Zaleski K. Enrile A. Weiss E. Wang X. (Eds.), Women’s journeys to empowerment in the 21st century: A transnational feminist analysis of women’s lives in modern times (pp. 289–307). Oxford University Press.

[bibr13-10778012231186814] Childress S. Gioia D. Campbell J. (2018). Women’s strategies for coping with the impacts of domestic violence in Kyrgyzstan: A grounded theory study. Social Work in Health Care, 57(3), 164–189. 10.1080/00981389.2017.1412379 29227740

[bibr14-10778012231186814] Childress S. Hanusa D. (2018). All the system is simply a soap bubble”: Legal help-seeking for domestic violence among women in Kyrgyzstan. Journal of Family Violence, 33(2), 147–160. 10.1007/s10896-017-9945-0

[bibr15-10778012231186814] Childress S. Panchanadeswaran S. Joshi M. (2021). Leaving and beyond: Voices of survivors of domestic violence from Kyrgyzstan. Journal of Interpersonal Violence, 36(3-4), 1718–1744. 10.1177/0886260517743550 29295004

[bibr16-10778012231186814] Childress S. Shrestha N. Anekwe K. Small E. McKay M. (2022). Service providers perspectives on barriers to help-seeking for intimate partner violence in Kyrgyzstan. Global Social Welfare, 9(3), 179–192. 10.1007/s40609-022-00226-x 37293550 PMC10249667

[bibr17-10778012231186814] Childress S. Shrestha N. Anekwe K. Wong M. Dudovitz R. (2023). Factors limiting institutional responses to domestic violence in Kyrgyzstan. Central Asian Survey, 42(2), 254–273. 10.1080/02634937.2022.2147146 37457923 PMC10348350

[bibr18-10778012231186814] Cowan C. P. Cowan P. A. Heming G. , et al. (2005). Two variations of a preventive intervention for couples: Effects on parents and children during the transition to school. In Cowan P. A. (Ed.), The family context of parenting in children’s adaptation to elementary school (pp. 277–312). Lawrence Erlbaum Associates. https://psycnet.apa.org/record/2005-04587-000 .

[bibr19-10778012231186814] Day T. (1995). The health-related costs of violence against women in Canada: the tip of the iceberg. Centre of Research on Violence Against Women and Children. http://www.crvawc.ca/section-research/publications_by_cat/p_publications_by_cat.html .

[bibr20-10778012231186814] DeKeseredy W. S. (2021). Bringing feminist sociological analyses of patriarchy back to the forefront of the study of woman abuse. Violence Against Women, 27(5), 621–638. 10.1177/1077801220958485 32956021

[bibr21-10778012231186814] Duvvury N. Callan A. Carney P. Raghavendra S. (2013, November 1). Intimate partner violence: Economic costs and implications for growth and development. Open Knowledge Repository. https://openknowledge.worldbank.org/handle/10986/16697 .

[bibr22-10778012231186814] Fernandez M. (2006). Cultural beliefs and domestic violence. Annals New York Academy of Sciences, 1087(1), 250–260. 10.1196/annals.1385.005 17189509

[bibr23-10778012231186814] Glaser B. G. Strauss A. L. (1967). The discovery of grounded theory: Strategies for qualitative research. Aldine.

[bibr24-10778012231186814] Heise L. (1998). Violence against women: An integrated, ecological framework. Violence Against Women, 4(3), 262–290. 10.1177/1077801298004003002 12296014

[bibr25-10778012231186814] Heise L. Pitanguy J. Germain A. (1994). *Violence against women: The hidden health Burden* (Report No. WDB255). http://www-wds.worldbank.org/external/default/WDSContentServer/WDSP/IB/1999/04/28/000009265_3970716144635/Rendered/PDF/multi0page.pdf.

[bibr26-10778012231186814] Human Rights Watch. (2017). *Kyrgyzstan: New domestic violence law—Government moves to improve response to abuse*. https://www.hrw.org/news/2017/05/10/kyrgyzstannew-domestic-violence-law.

[bibr27-10778012231186814] Kasturirangan A. Williams E. N. (2003). Counseling Latina battered women: A qualitative study of the Latina perspective. Journal of Multicultural Counseling and Development, 31(3), 162–178. 10.1002/j.2161-1912.2003.tb00541.x

[bibr28-10778012231186814] Kelly K. A. (2003). Domestic violence and the politics of privacy. Cornell University Press.

[bibr29-10778012231186814] Khalil U. Mookerjee S. (2019). Patrilocal residence and women’s social status: Evidence from South Asia. Economic Development and Cultural Change, 67(2), 401–438. 10.1086/697584

[bibr30-10778012231186814] Law of the Kyrgyz Republic on Aksakals (Elders) Courts. (2002). *Ministry of Justice of the Kyrgyz Republic*. No. 113. http://cbd.minjust.gov.kg/act/view/ru-ru/1070.

[bibr31-10778012231186814] Law of the Kyrgyz Republic on Protection from Domestic Violence. (2017). *Ministry of Justice of the Kyrgyz Republic*. No 63. http://cbd.minjust.gov.kg/act/preview/ru-ru/111570/15?mode=tekst.

[bibr32-10778012231186814] Law of the Kyrgyz Republic on Social and Legal Protection from Domestic Violence. (2003). Ministry of Justice of the Kyrgyz Republic. No. 62. http://cbd.minjust.gov.kg/act/view/ru-ru/1186.

[bibr33-10778012231186814] Law of the Kyrgyz Republic on Social Protection of the Population. (2001). *Ministry of Justice of the Kyrgyz Republic*. No. 111. http://cbd.minjust.gov.kg/act/view/ru-ru/943/80?cl=ru-ru.

[bibr34-10778012231186814] Lincoln Y. Guba E. (1985). Naturalistic inquiry. Sage Publications.

[bibr35-10778012231186814] McKay M. M. Gonzales J. J. Stone S. Ryland D. Kohner K. (1995). Multiple family therapy groups: A responsive intervention model for inner city families. Social Work with Groups, 18(4), 41–56. 10.1300/J009v18n04_04

[bibr36-10778012231186814] Ministry of Education and Science of the Kyrgyz Republic, UNICEF, & European Commission. (n.d.). *Who is a Social Pedagogue?* https://ffc.org.kg/images/soc_edu/sps.pdf .

[bibr37-10778012231186814] Muldoone R. Casabonne U. (2017). Gender norms in flux: Bride kidnapping and women’s civic participation in the Kyrgyz Republic. World Bank Group. http://hdl.handle.net/10986/28989 .

[bibr38-10778012231186814] National Statistical Committee, Ministry of Health of the Kyrgyz Republic, & ICF International. (2013). *Kyrgyz Republic Demographic and Health Survey*. Bishkek, Kyrgyz Republic, and Calverton, Maryland, USA: NSC, MOH, and ICF International. https://dhsprogram.com/pubs/pdf/fr283/fr283.pdf.

[bibr39-10778012231186814] National Statistical Committee of the Kyrgyz Republic. (2015). *Crime and public order in the Kyrgyz Republic: Statistical publication.* http://www.stat.kg/media/publicationarchive/e240e5ae-ffec-4acf-ad2f-ae3c66f8aef0.pdf .

[bibr40-10778012231186814] Nwokolo C. A. Shrestha P. N. Ferguson G. Shrestha B. Clark C. J. (2020). Contextual attributes of the family and community that encourage or hinder the practice of intimate partner violence in Nepal. South Asian Journal of Law, Policy, and Research, 1(1), 41–66. https://ssrn.com/abstract=3671493 .

[bibr41-10778012231186814] Rabbani F. Qureshi F. Rizvi N. (2008). Perspectives on domestic violence: Case study from Karachi, Pakistan. EMHJ-Eastern Mediterranean Health Journal, 14(2), 415–426. https://apps.who.int/iris/handle/10665/117454 . 18561735

[bibr42-10778012231186814] Raj A. Silverman J. (2002). Violence against immigrant women: The roles of culture, context, and legal immigrant status on intimate partner violence. Violence Against Women, 8(3), 367–398. 10.1177/10778010222183107

[bibr43-10778012231186814] Regulations on the procedure for identifying children and families in difficult life situations. (2015). *Decree of the Government of the Kyrgyz Republic*. №391. http://cbd.minjust.gov.kg/act/view/ru-ru/97689?cl=ru-ru.

[bibr44-10778012231186814] Rydstrom H. (2003). Encountering “Hot” anger: Domestic violence in contemporary Vietnam. Violence Against Women, 9(6), 676–697. 10.1177/1077801203009006004

[bibr45-10778012231186814] Schuler S. R. Islam F. (2008). Women’s acceptance of intimate partner violence within marriage in rural Bangladesh. Studies in Family Planning, 39(1), 49–58. 10.1111/j.1728-4465.2008.00150.x 18540523

[bibr46-10778012231186814] Ssewamala F. Bahar O. McKay M. (2022). Child behavioral health in Sub-Saharan Africa: Towards evidence generation and policy development. Springer Nature.

[bibr47-10778012231186814] Turaeva M. R. Becker C. M. (2022). Daughters-in-law and domestic violence: Patrilocal marriage in Tajikistan. Feminist Economics, 28(4), 60–88. 10.1080/13545701.2022.2060518

[bibr48-10778012231186814] United Nations Development Programme. (2013). *How much domestic violence costs in Kyrgyzstan?* https://www.kg.undp.org/content/kyrgyzstan/en/home/presscenter/articles/2013/12/17/how-much-domestic-violence-costs-in-kyrgyzstan-.html .

[bibr49-10778012231186814] United Nations Development Programme. (2013). *Human development reports*. https://hdr.undp.org/en/countries/profiles/KGZ.

[bibr50-10778012231186814] UN Women. (2017). *New progressive law on domestic violence is adopted in Kyrgyzstan.* https://www.unwomen.org/en/news/stories/2017/5/news-new-progressive-law-on-domestic-violence-adopted-in-kyrgyzstan .

[bibr51-10778012231186814] Walker C. (2020). Masculinity and gender-based violence in Eastern Europe and Central Asia: Engaging men in the gender equality agenda. The Bearr Trust. https://bearr.org/sharing/reports-and-articles/masculinity-and-gender-based-violence-in-eastern-europe-and-central-asia-engaging-men-in-the-gender-equality-agenda/ .

[bibr52-10778012231186814] World Health Organization. (2001). Putting women first: Ethical and safety recommendations for research on domestic violence against women. https://apps.who.int/iris/handle/10665/65893 .

[bibr53-10778012231186814] World Health Organization. (2005). WHO multi-country study on women’s health and intimate partner violence against women: Initial results on prevalence, health outcomes and women’s responses / authors: Claudia Garcia-Moreno et al. World Health Organization. https://apps.who.int/iris/handle/10665/43309 .

[bibr54-10778012231186814] World Health Organization. (2013). *Responding to intimate partner violence and sexual violence against women: WHO clinical and policy guidelines*. ISBN 9789241548595. https://apps.who.int/iris/bitstream/handle/10665/85240/9789241548595_eng.pdf.

[bibr55-10778012231186814] Yoshihama M. (2002). Breaking the web of abuse and silence: Voices of battered women in Japan. Social Work, 47(4), 389–400. 10.1093/sw/47.4.389 12450010

[bibr56-10778012231186814] Yoshioka M. R. Choi D. Y. (2005). Culture and interpersonal violence research: Paradigm shift to create a full continuum of domestic violence services. Journal of Interpersonal Violence, 20(4), 513–519. 10.1177/0886260504267758 15722509

